# Cystoscopic application of PuraStat^®^ in the treatment of radiation-induced haemorrhagic cystitis

**DOI:** 10.1308/rcsann.2023.0034

**Published:** 2024-02-16

**Authors:** H Darwazeh, L Hemsworth, L Smith, PC Ilie

**Affiliations:** ^1^ Norfolk and Norwich University Hospitals NHS Foundation Trust, UK; ^2^ The Queen Elizabeth Hospital King’s Lynn NHS Foundation Trust, UK; ^3^ Anglia Ruskin University, UK

**Keywords:** Refractory haematuria, Haemorrhagic cystitis, Radiation cystitis, PuraStat^®^

## Abstract

The use of radiotherapy has increased in recent years, especially for pelvic neoplasms, and this can result in long-term complications such as recurrent haemorrhagic radiation cystitis (RHC). A 73-year-old male patient presented to a hospital emergency department multiple times with visible haematuria and clots leading to urinary clot retention; he was finally diagnosed with RHC. During the last presentation, the bladder was irrigated continuously with saline using a three-way catheter. During hospitalisation, a cystourethroscopy was performed for bladder evaluation and clot evacuation. Multiple bleeding ulcers were recognised on the bladder wall, biopsies were taken for histopathology, and the ulcers cauterised. Packed red blood cell transfusions were required, and sodium hyaluronate (CystiStat^®^) bladder instillations were tried. There was no clinical improvement following any of these interventions. In light of the patient’s deteriorating condition, cystoscopic application of PuraStat^®^ 3ml was administered, which led to remission of the urinary bleeding in the short term. We continue to monitor the effects in the medium and long term. Based on current data, PuraStat^®^ haemostatic agent therapy may be considered for RHC, when traditional treatments are ineffective or infeasible, potentially eliminating the need for more aggressive therapy such as cystectomy.

## Background

Radiotherapy may be used alone or in conjunction with chemotherapy to treat pelvic neoplasms, such as prostate, bladder, uterus, ovary, cervix and rectal cancers. Toxic effects within genitourinary systems are likely to increase with higher radiation doses depending on the disease burden. Compared with other pelvic tissues, the urinary bladder is particularly sensitive to radiation doses at low levels.^1^ This may be attributed largely to the low turnover of urothelium cells.^2^

Reports have shown that 23% to 80% of all complications associated with pelvic radiation are related to recurrent haemorrhagic radiation cystitis (RHC), with severe haematuria occurring in 5%–8% of patients.^3^ RHC can occur as early as 3 months after radiotherapy, or as long as 14 years later. Studies have shown that males are much more susceptible to RHC than females (ratio of 2.8:1) simply because prostate cancer is strictly a male disease.^1,4^ It is believed that during RHC, mucosal arterioles and capillaries are obliterated by progressive fibrosis, resulting in hypoxia and subsequent necrosis of the urothelium.^3^ During cystoscopy, neovascularisation as well as telangiectasia are commonly observed, resulting in macroscopic haematuria that is severe and refractory.^1^ The consequences of excessive, recurrent and prolonged haematuria are reflected in high hospital costs and mortality rates, because successive transfusions and multiple hospitalisations are required.^5,6^

Anamnesis, combined with urine examination, urinary cytology and cystoscopy with biopsies are the most reliable means of confirming RHC as a clinical diagnosis. It is important to rule out upper urinary tract pathology and injury as a cause of haematuria using computed tomography (CT) and magnetic resonance imaging when a pelvic tumour has previously been diagnosed.^3^

A wide range of treatment options are available for RHC, including bladder irrigation, corticosteroids, oral oestrogen, intravesical treatment with alum or formalin and hyaluronic acid, sodium pentosan polysulfate, epsilon-aminocaproic acid, factor VIIa/factor VIII, hydrodistension of the bladder, embolisation or ligation of the internal vesical and iliac arteries, or, as a last resort, a cystectomy with urinary diversion may be recommended.^4^ Moreover, approximately 90% of patients with RHC benefit from hyperbaric oxygen therapy on a long-term basis, and there are no major side-effects affirmed.^7^ However, RHC remains a challenging condition for clinicians despite the availability of various treatment options, because it is often poorly treated in the short term, especially in the elderly.

## Case history

A 73-year-old male had undergone radiotherapy for prostate cancer: prostate-specific antigen at diagnosis 15.2ng/ml, (Gleason 4+3 bilateral) 10 years previously (in 2013).

**Figure 1:**
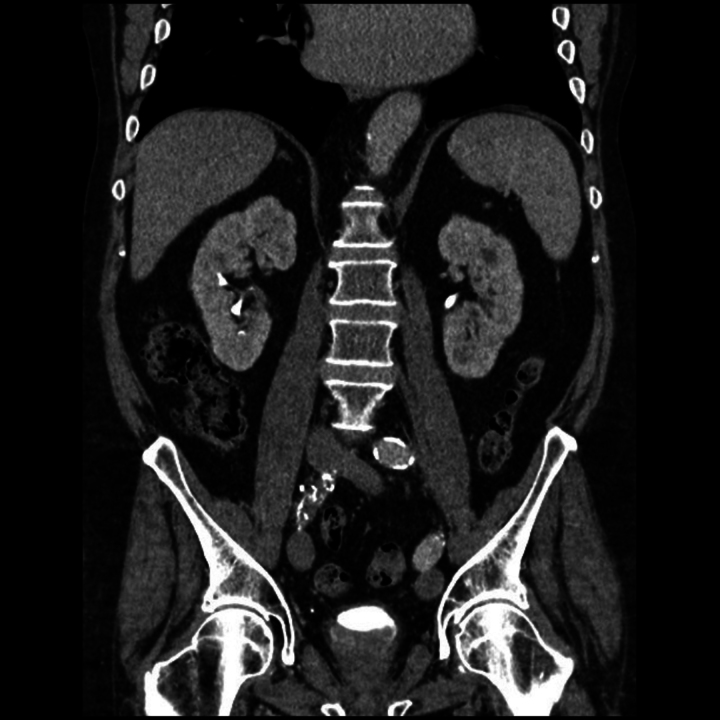
Computed tomography (CT) imaging of the abdomen and pelvis with contrast showing urinary bladder with slightly thickened walls and multiple clots; kidneys showing multiple bilateral small cortical cysts and no hydronephrosis

**Figure 2:**
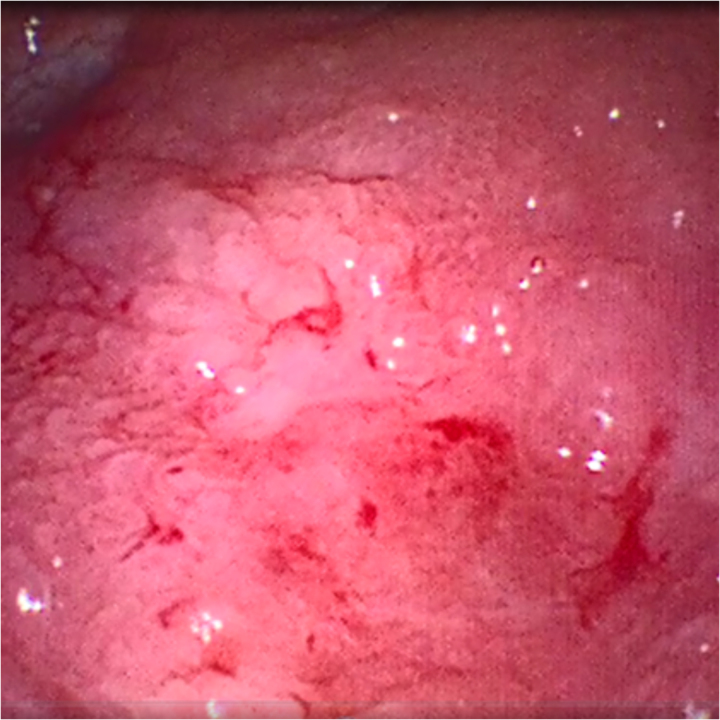
Image view of water irrigation flexible cystoscopy showing sites of bleeding and ulcerative lesions (neovascularisation) on the bladder wall

The patient required multiple packed red blood cell (RBC) transfusions after 9 years of radiotherapy owing to urinary clot retention and visible haematuria. During his most recent presentation, a three-way urinary catheter was inserted into the bladder, maintained on continuous saline irrigation and the patient was hospitalised in an in-patient unit. In admission laboratory tests, renal function and coagulation were: INR, 1.09; platelets, 194,000/μl; urea, 7.8mmol/l; creatinine, 123μmol/l; Na^+^, 133mEq/l; and K^+^, 3.7mEq/l. Haemoglobin (Hb) was found to be significantly reduced (68g/l) and C reactive protein (CRP) (80mg/l) was elevated. There were multiple bilateral small cortical cysts on the kidneys and no hydronephrosis on a CT scan of the abdomen and pelvis; the urinary bladder was distended, and the walls were slightly thickened, with multiple clots (Figure 1). We performed an emergency cystoscopy to evacuate clots and cauterise bleeding points in the bladder after prolonged gross haematuria. Multiple bladder biopsies were taken, and histopathological examination demonstrated inflammation, ulcer slough and scarring with no malignancy. RHC was diagnosed clinically, without evidence of other pathologies. Urine culture was positive for *Enterococcus faecalis* and treated with ciprofloxacin 500mg iv twice daily for 5 days. Hb level increased to 92g/l after administration of four packed RBC. The CRP level had reduced to 10mg/l after 20 days of admission, the urine culture showed no growth and the creatinine level had returned to baseline (89mmol/l). The patient was hemodynamically stable and accordingly discharged. During a 4-week period, bladder instillations of CystiStat^®^ (sodium hyaluronate 40mg) were initiated in an attempt to reduce prolonged haematuria. Because the patient continued to experience macroscopic haematuria, a water irrigation flexible cystoscopy was performed as an outpatient procedure to identify sites of bleeding and ulcerative lesions (neovascularisation) (Figure 2). Using sterile techniques and with full personal protective equipment, a flexible cystoscope was inserted through the urethra and into the bladder. Multiple aspirations of the bladder were conducted by connecting a 50ml syringe to the cystoscope port and the bladder was emptied completely. Using a new 50ml syringe, air was introduced repeatedly through the syringe until the bladder was partially full. Under cystoscope guidance, a syringe containing 3ml PuraStat^®^ was connected to a cystoscope port and 0.5ml of haemostatic agent was applied to each of the six ulcerative lesions. Distilled water (2ml) was added to the last two applications to clear the cystoscopic channel of the haemostatic agent. Finally, the cystoscopy was successfully retrieved after air was held in the bladder for 3 minutes to ensure haemostatic agents were properly attached to each lesion. This procedure led to the remission of urinary bleeding. The patient was scheduled for further PuraStat^®^ application once every 4 weeks.

## Discussion

Using self-assembling peptide technology, PuraStat^®^ gel is used for haemostasis in organ parenchyma, vascular anastomoses and small gastrointestinal blood vessels and capillaries. PuraStat^®^ has been reported to be a successful rescue therapy in the treatment of refractory acute gastrointestinal bleeding, following failure of two standard haemostatic methods.^8^ In this case report, remission of haematuria was achieved only following cystoscopic application of PuraStat^®^ haemostatic agent over bleeding ulcerative lesions on the urinary bladder wall.

Because of the similarity between activated PuraStat 3-D Matrix nanostructures and the natural extracellular matrix, as well as the known haemostatic effect of PuraStat^®^, it has been hypothesised that the activated PuraStat^®^ nanostructures will facilitate cell and tissue proliferation. As a result of scaffold material, cells and regenerative tissues will be able to adhere effectively, resulting in improved mucosal healing.^9,10^ In addition, PuraStat^®^ has been proven to be safe and effective in various surgical and endoscopic scenarios in both animal and human studies.^11^

## Conclusions

This case report can open the door for a new option for the management of RHC, because this medical device is already licensed for the management of radiation proctopathy. In addition, cystoscopic delivery of PuraStat^®^ is a simple technique that could be used widely. We acknowledge that this is a case report of using PuraStat^®^ in the management of RHC, and more evidence is needed to replicate the results we have observed. In the future, if our results are supported clinically by other studies, cystoscopic application of PuraStat^®^ could be recognised as one of the treatment options for RHC.

## Consent

Written informed consent was obtained from the patient for publication of this manuscript and accompanying images.
